# Efficacy of silver nanoparticles against *Trichinella spiralis* in mice and the role of multivitamin in alleviating its toxicity

**DOI:** 10.1038/s41598-024-56337-2

**Published:** 2024-03-10

**Authors:** Noha Madbouly Taha, Fady Sayed Youssef, Hend M. Auda, Mohamed M. El-Bahy, Reem M. Ramadan

**Affiliations:** 1 Department of Parasitology, Faculty of Medicine, Cairo University, Cairo, Egypt; 2 Department of Pharmacology, Faculty of Veterinary Medicine, Cairo University, Cairo, Egypt; 3 Department of Medicine and Infectious Diseases, Faculty of Veterinary Medicine, Cairo University, Giza, Egypt; 4 Department of Parasitology, Faculty of Veterinary Medicine, Cairo University, Cairo, Egypt

**Keywords:** *Trichinella spiralis*, Silver nanoparticles, Toxicity, Multivitamin, Drug discovery, Zoology

## Abstract

Trichinellosis is a worldwide zoonotic disease. The majority of currently available anti-trichinellosis medications exhibit inadequate efficacy. The efficacy of a natively prepared new formulation of silver nanoparticles (Ag-NPs) was evaluated in the treatment of *Trichinella spiralis* (*T. spiralis*) infection in mice alone and combined with multivitamin-mineral (MM). After investigating the product’s biological and pharmacological characteristics, its therapeutic dose was estimated to be Ag-NPs at 21.5 mg/kg B.W. This dose was orally inoculated to experimentally infected mice at 3–5 days post-inoculation (dpi) against the mature worms, at 8–10 dpi against the newborn larvae, and at 33–35th dpi against the encapsulated larvae. Each treatment’s efficacy was assessed by scarifying control and treated mice 3 days post-treatment. The drug alone or in supplement form has a high trichinocidal effect exceeding that of the reference drug. Early treatment (3–5 dpi) by Ag-NPs or Ag-NPs + MM and albendazole revealed high efficacy against the intestinal stage, reaching 93.3%, 94.7%, and 90.6% for the three treatments, respectively. The materials causing a significant (*P*-value < 0.001) decrease in the mean encapsulated larvae reached 86.61%, 89.07%, and 88.84%/gm of muscles using the three treatments, respectively. Moreover, all larvae extracted from Ag-NPs-treated groups failed to induce infection post-inoculation in new mice. Additionally, combining the material with MM proved to overcome the reversible adverse effects of silver material on the estimated redox parameters and liver and kidney biomarkers, denoting its ability to alleviate Ag-NP toxicity. In conclusion, the high trichinocidal effect of Ag-NPs against the adult and encapsulated larvae during a short inoculation period introduced Ag-NPs as an alternative to other nematicidal drugs.

## Introduction

Trichinellosis represents a worldwide health problem. The disease has been reported in 55 countries, or approximately 27.8% of the 198 world nations^1^. The distribution is mainly related to national eating behaviors, as trichinellosis infection occurs in humans by consuming raw or improperly cooked infected meat^2^.

Prolonged infection by *Trichinella* species in hosts causes a type of interaction between the parasite and the immune system of these hosts and appears in the form of a characteristic inflammatory reaction in the infected muscles^3^. In addition to the direct injury produced by the parasite, many other adverse effects are produced due to tissue damage, with oxidative stress emerging as the most dangerous reaction. This type of stress was expressed by an increase in the production of different stress markers, such as glutathione S-transferase omega-1 (GSTO-1), haem oxygenase I, and xanthine oxidase activity (X.O.)^4^, malondialdehyde (MDA), and reduced glutathione levels (GSH)^5^.

Most of the currently available trichinocidal drugs have many drawbacks, mainly because they are usually less effective against the encapsulated larvae in muscles. However, this may be due to resistance from recurrent application and low bioavailability, including these drug particles’ low tissue penetrating ability. For this reason, developing new effective formulations of drugs with high bio-viability properties for treating this parasite is still of high priority^6,7^.

The production of new formulations of some materials, like nanoforms, highly improved the original characteristics and effects of the products^8^. Ag-NPs have unique biological, physical, and chemical characteristics as they are small and have a large surface area. Moreover, they proved to have a broad spectrum of antimicrobial values against arthropods like *Rhipicephalus* ticks and mosquitoes’ larvae^9^ and protozoa such as *Giardia* species^10^. A locally produced new formulation of Ag-NPs was effectively devised and characterized by one of the present study’s authors. They characterized them by size, stability, and bioavailability^11^. Additionally, Taha et al.^12^ previously investigated this product of Ag-NPs in control of the adult *T. spiralis* worms in vitro and recorded 100% mortality in the exposed worms using 9.0 mg/ml for 24 h and 12 mg/ml for 12 h of exposure time. These doses induced irreversible changes in the tegument and body structures of the exposed worms inspected by scanning electron microscopy (SEM) and genotoxic damage in the dead parasite DNA measured by comet assay.

On the other hand, despite the highly promising efficacy of Ag-NPs as an antimicrobial or anti-parasitic agent, Gan et al.^13^ reported that silver as a material causes some drawbacks in the body, including subacute toxicity and severe oxidative damage in many organs of mice, with primary insults in the liver. For this reason, using drugs with antioxidant properties in combination with Ag-NPs for treating trichinellosis can protect the infected hosts from the suspected injurious oxidative stress produced by the parasite and the drug^14^.

There is an increasing interest in multivitamin-mineral (MM) supplements for prophylactic purposes. They succeeded in protecting against heart diseases, depression, cataracts, immune function disturbances, and cancer, and they induced protection against radiation^15^. MM supplementation, especially with vitamins E and C, possesses antioxidant properties and protects against pathological states caused by free radicals^16^.

The present study is a continuation of previous work that has been done on the same local, new formulation (Ag-NPs) as it was tested against *T. spiralis* adult worms in vitro^12^. In the first part of the present study, some pharmacological and biological properties of this new nanoformulation were investigated, including the determination of cytotoxicity and safety on human tissue culture cell lines. The second part evaluates the trichinocidal effect of Ag-NPs in *T. spiralis* experimentally infected mice. The material was supplemented with selected MM to overcome the known drawbacks of silver inoculation; however, its effect was investigated on the liver and kidney biomarkers in normal mice. The last step of this work includes examining the efficacy of Ag-NPs and Ag-NPs + MM against different stages of the parasite in experimentally infected mice. The value of supplementation by MM was investigated from the aspect of its ability to protect the inoculated mice from the oxidative stress of the parasite and improve the redox parameters in inoculated and control mice.

## Materials and methods

### Ethical approval

All procedures for handling mice and collecting samples were performed and approved under the relevant guidelines and regulations by the institutional animal care and use ethical committee of the Faculty of Veterinary Medicine, Cairo University (VET CU 01122022548). The study was carried out in compliance with the ARRIVE guidelines.

### Drug preparation (synthesis, doses, and toxicity)

#### Synthesis and formulation of silver nanoparticles (Ag-NPs)

##### Synthesis of Ag-NPs

Silver nanoparticle composite (Ag-NPs) was prepared in the laboratory as described in Taha et al.^12^ in the Pharmacology Department, Faculty of Veterinary Medicine, Cairo University, Egypt. The precursor materials are trisodium citrate and silver nitrate (Sigma, USA). The products were synthesized using the co-precipitation method. The product's final form is a water-soluble solution containing 100 ppm/ml of the active material^17^.

##### Characterization of the product

The morphological and physicochemical properties of the product were evaluated at Nawah Scientific, Egypt. Its shape and size were confirmed using a transmission electron microscope (TEM) (EM-2100 High-Resolution, Japan) at 20× magnification power and 200 kV. The shape of the Jol 2000 was evaluated using SEM. The Malvern, UK-made NanoSight NS500 was used to measure its size and zeta potential. Dynamic light scattering (DLS) was conducted in the size sector with the aid of Malvern Instruments Ltd. The produced Ag-NPs are spherical, less than 40 nm thick, with a 25 nm particle size and a 35 mV zeta potential. The used product is a water-soluble solution that contains 100 ppm/ml^12^.

#### Acute toxicity level and evaluation of the LD_50_ of Ag-NPs

Ag-NP lethal dose 50 (LD_50_) was determined according to Chinedu et al.^18^, where 5 separate groups (n = 5 mice), 20–25 g body weight (B.W.), were inoculated orally by the product at doses that ranged from 1000 to 5000 mg/kg B.W. Another corresponding group was left as naive control. The animals were still under observation for 3 days after inoculation. Mortality rate, toxicity symptoms, and post-mortem findings were recorded. LD_50_ of the tested drug was calculated using this formula: LD_50_ = D.M.−(∑AXB)/N.

#### Estimated therapeutic dose of the product in vivo

According to the obtained data on the drug toxicity and determination of the product LD_50_ (median lethal dose) and according to the Egyptian pharmacopeia^19^, Elkhawass et al.^20^ and Noaishi and Abd Alhafez^21^, the therapeutic dose of the drug was considered to be equal to 1/10 of the calculated LD_50_ (215 mg/kg B.W.).

#### Determination of Ag-NP cytotoxicity on HepG2 cells and human lung cells in vitro

The product’s cytotoxic effect was compared to crude silver against a liver cancer cell line (HepG2) in vitro, as previously described by Repetto et al.^22^. The standard neutral red cytotoxicity assay results were affected by the number of still-living cells in the tissue culture. The optical density of an extract of neutral red, measured by a spectrophotometer at 540 nm, was calculated using blank microtiter plates devoid of cells as a reference. The dose–response curves were created, and the concentration of the investigated substance that reflected a 50% inhibition of the uptake (IC_50_) was calculated.

As previously described by Repetto et al.^22^, for evaluating the effect of the product in vitro against a human lung cell line, the number of cells in the tissue culture affected the standard neutral red cytotoxicity assay results. In the present study, blank microtiter plates devoid of cells were used to measure the optical density of neutral red extract measured by a spectrophotometer at 540 nm as a reference for the test. The IC_50_ concentration of the investigated substance was calculated based on the dose–response curve.

#### Multivitamin-mineral supplement

The MM supplement (Abidec Advanced Multivitamin Syrup) was purchased from Omega Pharma Ltd. Components per 10 ml include 800 µg RE vitamin A, 7.5 µg vitamin D3, 2.1 mg a-TE vitamin E, 13.3 µg vitamin K, 64 mg vitamin C, 3 mg thiamin (vitamin B1), 2.40 mg riboflavin (vitamin B2), 16.67 mg N.E. niacin, 1.4 mg vitamin B6, 2.5 µg vitamin B12, 33.33 µg biotin, 2.81 mg pantothenic acid, and 20 mg 0.085 g per 100 g/100 kcal blackcurrant seed oil providing omega 6 and 9. Each mouse received 5 mg/kg/day orally using an oesophageal tube^23,24^.

#### Dose of the tested Ag-NPs and the MM supplement form

According to the previous toxicological investigation of this product and Elkhawass et al.^20^, the calculated LD_50_ of the material was 215 mg/kg. Following the guidelines provided by Egyptian Pharmacopeia^19^ and Noaishi and Abd Alhafez^21^, the material was administered as a trichinocidal at a rate of 21.5 mg/kg B.W. for three consecutive days, with the therapeutic dose being equivalent to 1/10 of the LD_50_.

To mitigate certain adverse effects of silver on animal health^13^, the material was applied in two forms; the first one is Ag-NPs, while the same dose was additionally tested in association with MM, as each mouse received 5 mg/kg/day (expressed as Ag-NPs + MM). The doses were calculated as described by Salama et al.^25^.

#### Investigating the effect of the materials on some organ biomarkers

For investigating the effect of the tested compounds (Ag-NPs, MM, and Ag-NPs + MM) on liver and kidney function, blood samples were collected from the mice sacrificed after 3 and 30 dpi by each material separately (5 per time of euthanasia). Samples were allowed to clot, and then their serum contents were separated by centrifugation at 3000 × g for 15 min. Sera were stored at − 20 °C till the time of biochemical assays. Biochemical assays, including urea, creatinine, AST, and ALT, were measured using commercial kits available in Egyptian laboratories^26^.

#### Reference drug

Albendazole 2.5% solution (produced by Pharma Swede) was used as a reference anti-helminthic drug at 50 mg/kg B.W. through the mouth. According to Jacob and Nair^27^, the dose was calculated by transforming the human dose of the drug into a mouse dose**.** The tested products and the reference drugs were administered orally to treated mice using an esophageal tube^23^.

### The in vivo study to assess the efficacy of drugs on *T. spiralis*-infected mice

#### Mice used in the study

Male and female laboratory-bred albino mice (n = 26–33), 8–10 weeks old, were included in the study. They were purchased from the rodent laboratory breeding unit in the Department of Animal Behavior, Faculty of Veterinary Medicine, Cairo University, Giza, Egypt. The mice were left for acclimatization for one week before being included in the study groups. Mice were still under standard rearing conditions, drinking clean water ad libitum, and fed a commercial rodent diet.

#### *T. spiralis* strain and oral inoculation of mice

**Figure 1:**
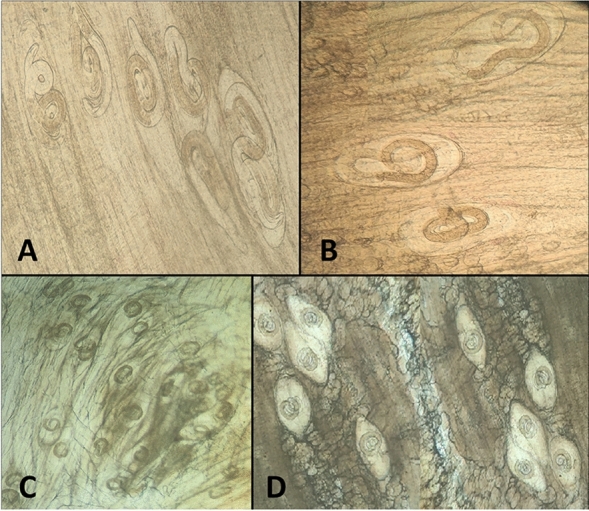
(**A**) Early migrating larvae of *T. spiralis* in the muscles of pretreated mice at 8th dpi, showing the early incomplete reaction around the larvae. (**B**) large cyst area with thin tissue reaction around the larvae (trichinoscope compressed). (**C**) More developed cysts with marked tissue reactions in the experimentally infected mice at 33 dpi. D. *T. spiralis* cysts in the muscles of pigs were used in experimental infection of different mouse groups.

*Trichinella* species larvae used during this study (Fig. 1) were extracted from naturally infected pigs' diaphragmatic muscles directly after slaughtering at a Cairo abattoir in Egypt. After inspection of suspected samples by trichinoscope, heavily infected pieces were minced, and then their larvae were extracted using the digestion technique described by Khalifa et al.^28^. In brief, the macerated minced muscles were transferred to a ten-time volume of digestion solution composed of 1% pepsin HCl in 200 ml of distilled water and then incubated at 37 °C for one hour with continuous stirring. Larvae liberated in the solution were collected after sedimentation, washed two times in phosphate-buffered saline PBS (pH—7.4), and kept in enough PBS. A representative number of these collected larvae were identified morphologically according to Mayer-Scholl et al.^29^ from aspects of their size using a micrometer slide and eyepiece, the form of their cuticles, and their characteristic rounded posterior ends without any appendages, shape, or length of their esophagus and its stichocyte compartments. The number of larvae per one ml of the final solution was determined microscopically using the McMaster counting chambers according to the guidelines of the International Quality Assurance for Diagnostic Laboratories^30^. These larvae were enlisted in additional molecular studies conducted by the same authors. During this process, they were genotyped after PCR and sequence of the COXI locus; they were determined to be *T. spiralis*, and the sequence obtained was deposited in the GenBank under the accession number OR271983. The clean, active motile larvae were used in oral inoculation of different mouse groups enrolled in the study at 200 larvae/mouse doses using a suitable stomach tube, according to El Temsahy et al.^31^.

#### Experimental design

**Figure 2:**
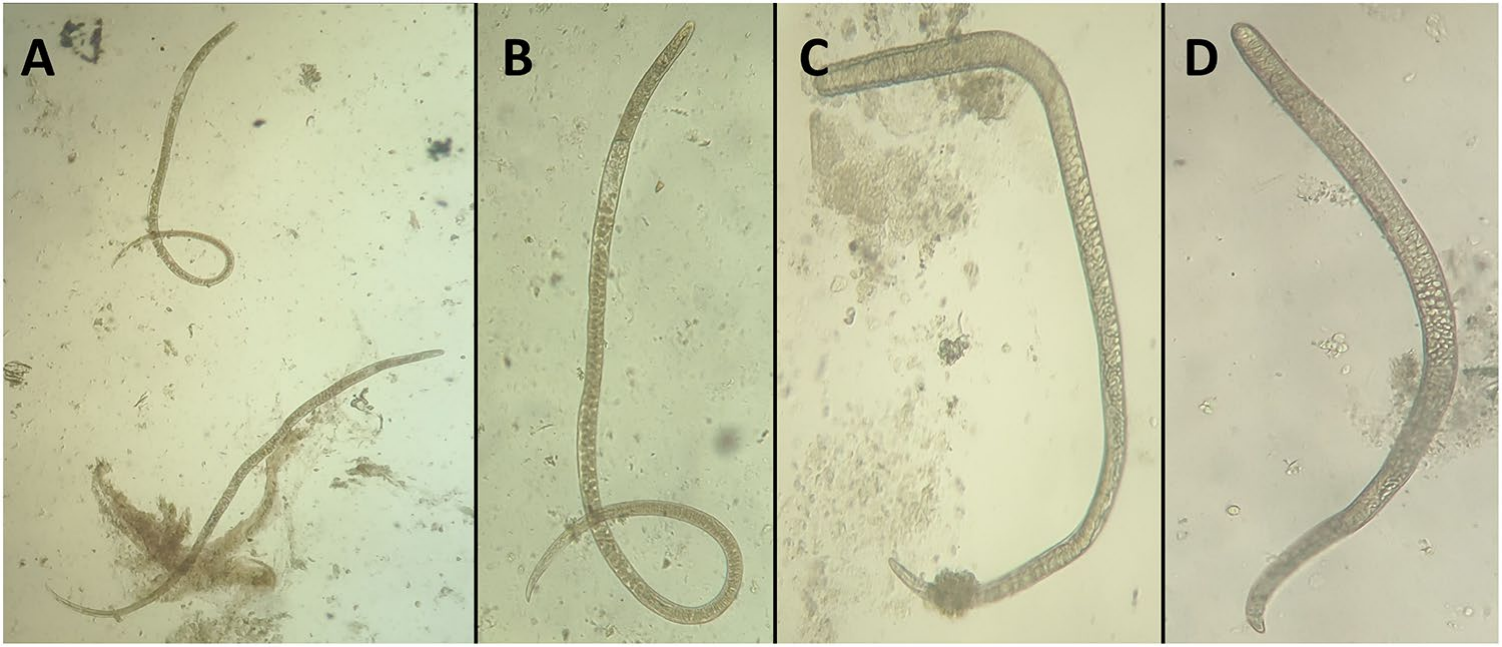
(**A**) and (**B**) *Trichinella spiralis* worms that were detected in the intestines of mice at time of first treatment (3rd dpi); (**C**) and (**D**) old adult worms that were detected in the intestines of untreated mice at 8th dpi. The figure shows the variation in size and degree of development.

The present study aimed to test the efficacy of treatment with Ag-NPs and its supplemented formulation against different stages of *T. spiralis* in experimentally infected mice. The selected time of therapy against the target stages of the parasite (intestinal stage, migrating larvae in the body tissue, and newborn and encapsulated larvae in muscles) was selected as previously determined by Yadav and Temjenmongla^32^ for three successive days in each time chosen as follows: Mice in G-I were treated at 3rd, 4th, and 5th dpi for testing the effect of treatment on the adult intestinal stage; mice in G-II were treated at 8th, 9th, and 10th dpi to attack the remaining adult in the intestine and the newborn migrating larvae; and those in G-III were treated at 33rd, 34th, and 35th dpi to evaluate the efficacy of the material against the encapsulated larvae in muscles. All mice enrolled in the experiment except the control non-exposed groups were orally inoculated by 200 larvae/mouse. Mice were divided into three main groups (G-I, G-II, and G-III), with 35 mice in each group. After this, each group was divided into five subgroups (7 mice each). The first subgroup was treated by Ag-NPs at 21.5 mg/kg. The second subgroup was treated by Ag-NPs at the same dose combined with MM syrup 5 mg/kg. The third subgroup was treated with the reference drug (albendazole, 50 mg/kg); the fourth was left as an infected non-treated control; and the fifth was the negative control. Mice in G-I were treated at 3rd, 4th, and 5th dpi, and those in G-II were treated at 7th, 8th, and 9th dpi. Mice in G-III were treated at 33rd, 34th, and 35th dpi. Two mice from each sub-group were euthanized by isoflurane inhalation (5%) before each treatment to investigate the condition of infection in the intestine and encapsulate larvae in muscles before the application of the selected treatment^33^. In each case, all mice in the treated and control groups were euthanized after 3 days following the last day of treatment. After all mice in the same group were sacrificed, their small intestines were opened in warm PBS; all the present worms were extracted and counted (Fig. 2)^12^. Additionally, pieces from the body and diaphragmatic muscles were compressed by trichinoscope plates and investigated microscopically to determine the stage of development of the cyst and the presence of early migrating larvae (Fig. 1)^28^. After removing the skin, internal organs, terminal parts of the feet, and the mouse heads of infected and control mice, the carcasses were weighed and then dissected into small pieces and minced before transferring the whole tissue to the digestion process. At the end of the digestion process, all liberated larvae in the solution were collected by sedimentation, washed in PBS (pH 7.4), identified, and counted, and the number of larvae/gm in whole samples was calculated as before^32,34^. The motility of the collected larvae per separate treatment was investigated under the microscope, while their infectivity was tested by oral inoculation of 5 new mice per trial (200 larvae/mouse).

The treatment efficacy was calculated from the reduction rate in the mean number of collected parasites from the treated mice compared to that obtained from the control infected non-treated mice, which was calculated using the equation.

#### Measuring oxidant/antioxidant status in mouse tissue homogenates (redox parameters)

The changes in the oxidant/antioxidant status were evaluated in the infected tissues to assess the result of the parasite and the value of different treatment regimens in improving some redox parameters. Pieces from the intestines and muscles of control and infected untreated and treated mice were cut out, washed with cold saline, dissected into small pieces, and weighed before homogenizing them in five volumes of PBS, pH 7.4. The supernatant solution was collected after centrifugation at 4 °C at 10,000 × g for 20 min, allocated in a 1 ml vial, and frozen at − 80 °C until use. The Bradford method^35^ was used to determine its protein contents with bovine serum albumin as a standard. The total antioxidant capacity (TAC) levels were measured in the homogenate extract of intestines, and muscles were analyzed using the enzymatic colorimetric method using kits available from a biodiagnostic company in Egypt. Additionally, malondialdehyde (MDA) levels were measured using commercial kits (Biodiagnostic, Egypt), as previously described by Othman et al.^36^.

### Statistical analysis

The data were displayed as mean ± standard deviation with the SPSS 27 (IBM, NY, USA) program, and the data were evaluated statistically using an ANOVA test; however, the differences were considered significant at *P*-values ≤ 0.001^37,38^.

## Results

### Acute toxicity level and evaluation of the LD_50_ of Ag-NPs

Results in Table 1 showed that the calculated LD_50_ of the prepared Ag-NPs was 215 mg/kg B.W. Considering the previous basis for calculating a therapeutic dose of the product estimated at 1/10 of the obtained LD_50,_ the advisable therapeutic dose of this form of Ag-NPs was 21.5 mg/kg B.W.

**Table 1:** Determination of the LD_50_ of nanosilver in mice (n = 5).

Dose in mg/kg B.W	No. of dead animals in each group	A	B	A × B	Σ (A × B)	Dm	Calculated Ld_50_
100	0/5	0	50	0	425	300	Dm−**Σ (A** × **B)**/N 300–425/5 = 215 mg/kg B.W
150	1/5	0.5	50	25			
200	2/5	1.5	50	75			
250	3/5	2.5	50	125			
300	5/5	4	50	200			

DM, the highest dose that kills all inoculated mice; A, mean of dead mice between 2 successive groups; B, constant factor between 2 successive doses; N, number of mice per group. Σ = Sum of (A × B).

### Cytotoxic effect of Ag-NPs on HepG2 and human lung cell line

Testing the level of cytotoxicity of Ag-NPs on the liver cancer cells (HepG2) and human lung normal cell line in vitro in comparison with that of crude silver revealed that the nanoform has a marked inhibiting effect on the multiplication of both types of cells at the lower half maximal inhibitory concentration (IC_50_) recorded using a dose reached to 23 µg/ml and 12.5 µg/ml for the human lung normal cell line and HepG2, respectively, while it was higher as 150 µg/ml and 187 µg/ml for the crude silver against both types of cells, respectively, using the colorimetric assays.

### Effect of treatment on serum biomarkers

In vivo, evaluation of the adverse effects of the tested two forms of the product (Ag-NPs and Ag-NPs + MM) at the calculated therapeutic dose (21.5 mg/kg B.W.) on liver and kidney functions and in control non-infected mice is demonstrated in the Tables 2 and 3. Ag-NPs induced a significant alteration (*P-*value < 0.001) compared with the control non-inoculated mice on the estimated parameters of both organs. Inoculation of mice by MM alone at this time did not show elevation of liver and kidney function parameters, with no significant difference from that of the control non-inoculated mice (Tables 2 and 3). Inoculation of mice by MM alone or in combination with the Ag-NPs showed substantial (*P-*value < 0.001) improvement in the enzyme levels compared to the Ag-NPs-treated and control groups. Re-evaluation of these parameters at 30 days post-inculcation revealed that all of the above alterations are considered reversible as there was marked improvement in all parameters.

**Table 2:** Variations in liver function enzymes of mice after inoculation by Ag-NPs (n = 5).

Inoculated materials	At 3 days post inoculation		At 30 days post inoculation	
	ALT (U/ML)	AST (U/ML)	ALT (U/ML)	AST (U/ML)
Ag-NPs at 21.5 mg/kg. B.W	47.60 ± 1.96^c^	155.44 ± 2.38^c^	34.28 ± 1.81^b^	127.16 ± 2.16^b^
Ag-NPs + MM	36.34 ± 1.35^b^	127.60 ± 1.65^b^	32.92 ± 1.06^a^	122.08 ± 2.42^ab^
MM at 5 mg/kg. B.W	30.56 ± 1.48^a^	114.16 ± 2.47^a^	29.42 ± 1.69^a^	112.52 ± 2.08^a^
Control non-inoculated mice	31.06 ± 1.13^a^	118.02 ± 2.82^a^	30.38 ± 1.46^a^	113.96 ± 2.28^a^

Column of the different letters is significant difference *P*-value < 0.001.

ALT, alanine transaminase; AST, aspartate aminotransferase.

**Table 3:** Variations in some kidney Function parameters of mice after inoculation by Ag-NPs (n = 5).

Inoculated materials	At 3 days post inoculation		At 30 days post inoculation	
	Urea (mg/dl)	Creatinine (mg/dl)	Urea (mg/dl)	Creatinine (mg/dl)
Ag-NPs at 21.5 mg/kg. B.W	62.70 ± 1.95^c^	2.70 ± 0.16^b^	49.04 ± 1.07^b^	1.91 ± 0.12^ab^
Ag-NPs + MM	51.46 ± 1.43^b^	2.10 ± 0.15^ab^	47.52 ± 1.49^ab^	1.78 ± 0.08^ab^
MM at 5 mg/kg. B.W	43.74 ± 0.9^a^	1.40 ± 0.16^a^	45.98 ± 1.48^a^	1.59 ± 0.06^a^
Control non-inoculated mice	46.60 ± 1.12^a^	1.38 ± 0.19^a^	46.44 ± 1.19^a^	1.53 ± 0.08^a^

Value per column of the different letters is a significant difference *P*-value < 0.001.

### Efficacy of Ag-NP treatment on adult *T. spiralis* worms in the intestine

The treatment of mice early infected with *T. spiralis* (G-I) using 21.5 mg/kg B.W. of Ag-NPs or supplementation with MM (Ag-NPs + MM), in comparison with albendazole at 50 mg/kg B.W. as the reference drug, administered at 3rd, 4th, and 5th days post-infection and confirmed 3 days post-treatment, resulted in a substantial reduction (*P*-value < 0.001) in the mean number of recovered adult worms. The counts reached 2 ± 0.4, 1.6 ± 0.4, and 2.8 ± 0.8 in the groups treated with the drugs mentioned above, respectively, as opposed to 30.2 ± 3.5 in the untreated control group. This denotes an efficacy, represented by the worm reduction rate, of 93.3%, 94.7%, and 90.6% for the three drugs, respectively. Adding MM to the Ag-NPs improved the efficacy of the tested nanoproduct but without a significant difference between the efficacy of the three tested drugs (Table 4). Inspection of the intestines representing samples from these mice at 8th–10th dpi revealed an overall reduction in adult worms before treatment application ranging from 8 to 10 worms/mice. Application of the same previous treatment as in G-II showed an efficiency of 87.5%, 88.6%, and 86.3% for the above three drugs, respectively, with more superiority for the Ag-NPs + MM than the other two drugs but with no significant difference. Inspection of infected mice treated at 33th–35th dpi (G-III) showed no isolated active worms from the intestines, except 2–5 worms recovered from the intestines of the control untreated group (Table 4).

**Table 4:** Mean number of adult *T. spiralis* worms collected from each euthanized mouse group post different regimens of treatment (n = 5).

Treated and control Group of mice		Dose of Treatment	(Mean ± SD)	Treatment efficacy
Group (I)	Infected Treated with Ag-NPs at 3rd, 4th and 5th dpi	21.5 mg/kg	2 ± 0.4*	93.3%
		21.5 mg/kg + MM 5 mg/kg	1.6 ± 0.4*	94.7%
	Albendazole 50 mg/kg		2.8 ± 0.8*	90.6%
	Infected untreated mice		30.2 ± 3.5	–
Group (II)	Infected Treated with Ag-NPs at 8th, 9th and 10th dpi	21.5 mg/kg	1.1 ± 0.3*	87.5%
		21.5 mg/kg + MM 5 mg/kg	1 ± 0.7*	88.6%
	Albendazole 50 mg/kg		1.2 ± 0.4*	86.36%
	Infected untreated mice		8.8 ± 0.8	–
Group (III)	Infected Treated with Ag-NPs at 33rd, 34th and 35th dpi	21.5 mg/kg	0	–
		21.5 mg/kg + MM 5 mg/kg	0	–
	Albendazole 50 mg/kg		0	–
	Infected untreated mice		3.4 ± 1.1	–
Group (IV)	Control non-infected mice		0	–

*P-value < 0.001 statistically significant.

### Efficacy of Ag-NPs on newborn and encapsulated *T. spiralis* larvae in tissues

In testing the product's efficacy against *T. spiralis*, encysted larvae showed marked efficacy for Ag-NPs + MM compared to the other tested compound. Treatment at 3th–5th dpi (G-I) caused a significant decrease in the cyst intensity in muscles per mouse, reaching 86.61%, 89.07%, and 88.84% using Ag-NPs, Ag-NPs + MM, and albendazole, respectively. This efficacy was associated with a significant decrease (*P*-value < 0.001) in the mean number of collected larvae from the muscles of these mice compared to the larvae diagnosed in the infected, non-treated mice. None of the isolated larvae at this stage of parasite development (3rd–5th dpi) successfully infected newly inoculated mice (Table 5).

**Table 5:** Mean number of encysted *T. spiralis* larvae/one gram of mouse muscle when euthanized post each different regime of treatment (mean body weight = 32 g and n = 5).

Treated & control Group of mice		Dose of treatment	Mean ± SD	Treatment efficacy	The infectivity of the cyst
Group (I)	Infected Treated with Ag-NPs at 3rd, 4th and 5th dpi	21.5 mg/kg	45 ± 6.7*	86.61%	No
		21.5 mg/kg + MM 5 mg/kg	36.8 ± 5.45*	89.07%	No
	Albendazole 50 mg/kg		37.6 ± 4.97*	88.84%	No
	Infected untreated mice		336.8 ± 13.84	-	No
Group (II)	Infected Treated with Ag-NPs at 8th, 9th and 10th dpi	21.5 mg/kg	119.6 ± 7.16*	70.77%	Dead
		21.5 mg/kg + MM 5 mg/kg	105.2 ± 6.43*	74.29%	Dead
	Albendazole 50 mg/kg		111.4 ± 5.94*	72.78%	Dead
	Infected untreated mice		409.2 ± 25.59	-	Infective
Group (III)	Infected Treated with Ag-NPs at 33rd, 34th and 35th dpi	21.5 mg/kg	255.2 ± 28.33*	55.73%	Not infective
		21.5 mg/kg + MM 5 mg/kg	243.6 ± 16.52*	57.81%	Not infective
	Albendazole 50 mg/kg		268.8 ± 30.87*	53.47%	Infective
	Infected untreated mice		576.4 ± 42.16	-	Infective
Group (IV)	Control non-infected mice		0	0	No

*P-value < 0.001 statistically significant.

With the delay of treatment until 8th–10th dpi (G-II), the recorded number of encysted larvae in muscles increased in all groups, while the application of therapy caused a significant reduction (*P*-value < 0.001) in the mean number of extracted cysts from treated groups in comparison with the control untreated group. The obtained data demonstrated lower efficacy against the encysted larvae in muscles than that recorded in G-I, which was treated early at 3rd–5th dpi. The efficacy was 70.77%, 74.29%, and 72.78% in mice treated with the three tested drugs. It was essential to determine that larvae extracted from treated groups at this time post-infection (8–10 dpi) are weak or nonmotile and approximately dead, while those obtained from untreated control groups are live and motile and able to induce infection in newly inoculated mice (Table 5).

Testing the efficacy of nanoproduct in both forms against the late encapsulated larvae (G-III) by treatment of infected mice at 33rd–35th dpi showed a significant reduction in the mean number of the encysted larvae from 576.4 ± 42.16 in the untreated group to 255.2 ± 28.33, 243.6 ± 16.52, and 268.8 ± 30.87 in mice treated with 21.5 mg/kg Ag-NPs, 21.5 mg/kg Ag-NPs + MM, and 50 mg/kg albendazole, respectively. It was essential to determine that the extracted larvae from the muscles of the groups treated by both forms of Ag-NPs at 21.5 mg/kg B.W. can only infect newly inoculated mice. In contrast, the larvae extracted from the groups treated with albendazole and those from untreated control mice induced infection in newly inoculated mice (Table 5). There was no significant difference in the efficacy between the three drugs used.

### Effect of treatment on redox parameters in intestinal and muscle homogenates

Estimating the efficacy of Ag-NPs on two primary redox markers (MDA levels and TAC) in the homogenate tissues of small intestines and muscles of mice infected and treated by the two forms of Ag-NPs in comparison with the infected, untreated, and control, non-infected mice is described in Table 6. Infection by *T. spiralis* altered the estimated parameters’ values compared to the non-infected control and was higher in mice infected and treated by Ag-NPs alone. Supplementing the nanoparticles with MM significantly improved the condition.

**Table 6:** Variations in mean values of redox parameters in skeletal muscle and small intestine homogenates of treated and control mice (n = 5).

Group of mice	Skeletal muscle homogenates in Group (III) treated at 33–35th dpi		Intestinal homogenates in G(I) treated at 3–5th dpi	
	MDA (nmol/g tissue)	TAC (µmol/g tissue)	MDA (nmol/g tissue)	TAC (µmol/g tissue)
Infected Treated with Ag-NPs	6.30 ± 0.09*	2.52 ± 0.11*	5.31 ± 0.07*	1.63 ± 0.12*
Infected Treated with Ag-NPs + MM	4.16 ± 0.10*^A^	3.19 ± 0.12*^A^	3.04 ± 0.13*^A^	2.75 ± 0.14*^A^
Infected untreated	4.71 ± 0.06*^A^	2.87 ± 0.08*^A^	3.20 ± 0.09*^A^	2.37 ± 0.28*^A^
Control non-infected mice	2.65 ± 0.09	5.53 ± 0.08	1.61 ± 0.16	4.54 ± 0.17

MDA, level of Malondialdehyde (nmol/g tissue); TAC, total antioxidant capacity (µmol/g tissue).

*P-value < 0.001 compared to the infected nontreated group.

^A^*P*-value < 0.001 compared to the group treated with Ag-NPs.

## Discussion

Trichinellosis is a zooanthroponosis that affects approximately 11 million individuals worldwide^34^. Some issues support disease persistence, including improperly inspected pig meat in abattoirs, poor hygienic standards, and ineffective medications for trichinellosis treatment, especially against the muscle stages of the parasite with developing drug resistance. The need for a new, alternative, and effective drug against *T. spiralis* has been aroused^36^.

Nanotechnology is a promising era for developing effective de novo materials with dimensions ranging from 1.0 to 100 nm^39^. In this study, a new formulation of Ag-NPs that is highly purified was newly synthesized in the Department of Pharmacology, Faculty of Veterinary Medicine, Cairo University, by a simple low-cost sonochemical method. The Ag-NPs were characterized using SEM and TEM imaging; however, they were confirmed to have suitable dimensions and spherical form. Zeta size was measured at 25 nm, while zeta potential was evaluated at 35 mV. Recently, the data obtained by Youssef et al.^11^ agree with these findings.

The antioxidant combination with the parasiticidal medications potentiates the host's immune response, and deficient immunity additionally affects the release of reactive oxygen species (ROS) that suppress cell division and growth. Combining the specific drugs with antioxidants is a safer and more effective alternative to higher doses. In this study, nanomaterials’ heightened effectiveness, compared to other products supported by various researchers, is attributed to their distinctive and advanced physicochemical properties. Nanomaterials have a huge surface-to-volume ratio, high reactivity, stability, measured molecular sizes, effective bioactivity, and high bioavailability, as well as controlled delivery of loaded drugs to the target site^12,28^.

This study agreed with previous works that showed the production of silver in nanoform highly improved its cytotoxic potential effects against cancer and normal cultured cell lines. The produced Ag-NP formulation inhibited the liver cancer cell's proliferation in vitro. However, this was in agreement with Padmini et al.^40^, who synthesized Ag NPs with sizes ranging from 20 to 40 nm. Its products showed significant cytotoxic effects against A549 human lung cancer cells at an IC_50_ of 22 μg/ml. In addition, Faedmaleki et al.^41^ studied the cytotoxicity of nanosilver on HepG2 and reported that Ag-NPs caused a concentration-dependent decrease in cell viability in both cells. An IC_50_ value of 2.764 ppm (µg/ml) was calculated in the HepG2 cell line, and an IC_50_ value of 121.7 ppm (µg/ml) was calculated in the primary liver cells of mice. However, they additionally showed that Ag-Nps had cytotoxic effects on the HepG2 cell line and primary liver cells of mice. Moreover, Ag-Nps had a 44-fold stronger inhibitory effect on the growth of cancer cells (HepG2 cell line) than normal cells (primary liver cells of mice). However, the superiority of the nanoform of silver was related to the especially known character of nanoparticles concerning their minute size and high penetrating ability to different cell membranes^12^.

In the present study, the locally prepared Ag-NPs were administered in vivo in mice for the first time due to previous knowledge about the adverse effect of Ag on the function of body organs as described by Kim et al.^42^ and Tang et al.^43^, who stated that oral inoculation of Ag-NPs disseminates through circulation and is mainly detoxified in the liver, resulting in marked changes in hepatic enzymes, hepatotoxicity, and renal toxicity that were reported even by inhalation or subcutaneous administration. Additionally, compounds containing N.P.s were stated to have in vivo toxic effects mainly through oxidative stress produced by lipid peroxidation^44^. Moreover, *Trichnilla* infection causes significantly elevated liver enzymes (AST and ALT). This elevation might be related to hepatic damage caused by the migrating larvae or the toxic metabolic products of the parasite^45–47^. For the above reasons, the present study tended to evaluate the adverse effect of the tested Ag-NPs on the organ functions in control non-infected mice to avoid interaction in the impact due to the parasite and the materials.

Moreover, the treatment was applied for a short period (3 days only). Supplementing the Ag-NPs by MM is trying to overcome the adverse effect of the nanomaterial and following up on the changes for the 30th-day post-inoculation. The results obtained from this study demonstrate that oral inoculation of non-infected mice with the calculated therapeutic dose (21.5 mg/kg B.W.) of the two tested product forms (Ag-NPs and Ag-NPs + MM) induced alterations in liver and kidney biomarker parameters when compared to the control group of non-inoculated mice. These changes were observed three days post-inoculation on the assessed parameters for both organs. The lustration was more significant in groups inoculated by Ag-NPs alone than in those supplemented with MM. The inoculation of mice by MM alone improved the estimated liver and kidney function parameters without significant differences from those of the control non-inoculated mice. Inoculation of mice by MM alone or in combination with the Ag-NPs showed substantial (*P*-value < 0.001) improvement in the enzyme levels compared to the Ag-NPs-treated and control groups. All recorded alterations under this experiment's short administration period (3 days) appear reversible as the condition returned to semi-normal status with an insignificant difference from the control when re-estimated in the same mouse groups at 30th dpi. It is essential to mention that MM administration highly improved all of these markers over those in the control uninoculated groups.

The significant increase in AST and ALT levels in Ag-NPs-treated groups was related to other groups. However, this might be due to the toxic effect of Ag-NPs on the liver, as reported by Nakkala et al.^48^, who stated that Ag-NPs led to histopathological damage in the liver mainly in the form of an inflammatory reaction with accumulation of inflammatory cells, necrosis, fibrosis, and congestion. The results of this work are in agreement with Lee et al.^49^, El Mahdy et al.^50^ and Ansar et al.^51^.

In this study, the adverse effect of these Ag-NPs was affected mainly by the size of the particles as well as the length of the administration period. However, this agrees with Cho et al.^52^, who mentioned that intraperitoneal administration of small-sized Ag-NPs (10 nm) increased only AST from increasing tendency in ALT. Additionally, revealed that AST was elevated only in rats after oral administration of Ag-NPs (1 mg/kg) for 28 days, with only minor pathological alterations in the liver and kidneys. However, Pourhamzeh et al.^53^ did not state any considerable elevation in the AST and ALT levels following oral administration of Ag-NPs in rats, and these results might be due to the large size of Ag-NPs used (78.59 nm). However, this was contrary to Al‐Doaiss et al.^54^, who mentioned that the small size of Ag-NPs (10 nm) could be more injurious to hepatic tissues than the larger size of Ag-NPs. In addition, Sarhan et al.^55^ concluded that Ag-NP concentrations could alter organ damage. In addition, using biodegradable and biocompatible organic elements like polymers or lipids as a capsule for Ag-NPs could protect tissues from toxic damage.

In the current study, the impact of MM administration in alleviating the toxic effects produced by Ag-NPs was evaluated. The results showed that combined administration of MM with Ag-NPs significantly reduced serum AST and ALT levels (*P*-value < 0.001). However, these results agree with Hasan et al.^23^, who revealed that MM brought all stress markers' parameters to normal levels in tested mice after inducing chronic unpredictable oxidative stress. Furthermore, Namazi et al.^56^ have highlighted that combining an antioxidant with Ag-NPs is essential to mitigate its potential toxicity and optimize the utilization of its high efficacy. Penicillamine was added to Ag-NPs and improved all biochemical parameters, tissue damage, and the extent of oxidative stress produced in mice by Ag-NPs.

The current study was designed to test the efficacy of Ag-NPs against *T. spiralis* in different life stages by administering them at known times post-infection, following the schedule previously illustrated by Yadav and Temjenmongla^32^. Testing was done against the adult worms at the 3rd dpi and against newborn larvae at the 8th dpi against the encapsulated ones after 33 dpi. In this study and on trial, however, it was recommended that other medications be used in conjunction with the tested drugs to prevent drug interactions with those described by the same author, which involves eliminating unintended phases.

As this formulation of Ag-NPs is in vivo inoculated for the first time, the study was directed to determine its toxic dose experimentally. After calculations of its LD_50_ according to the methods of Chinedu et al.^18^ and Salama et al.^25^, who proved that LD_50_ was 215 mg/kg B.W. and according to Egyptian pharmacopeia^19^, Elkhawass et al.^20^, and Noaishi and Abd Alhafez^21^, who recommend the therapeutic dose as 1/10 of LC50, the study determined the dose of 21.5 mg/kg B.W. is an advisable therapeutic dose for this new product.

Testing the efficacy of Ag-NPs after supplementing the material with MM against different stages of *T. spiralis* in experimentally infected mice for 3 successive days revealed marked efficacy for the form supplemented with MM compared to the Ag-NPs alone and the reference drug (albendazole). Oral inoculation by the three materials caused a significant decrease (*P*-value < 0.001) in the mean number of adult *T. spiralis* worms in the intestines compared to the infected untreated control group, with percentage reductions of 93.3%, 94.7%, and 90.6%, respectively, with no significant difference observed between both groups. This effect decreased to 87.5%, 88.6%, and 86.36% in groups treated at 8th–10th dpi. The decrease in the efficacy rate of the drugs was related to the complete absence of the worms from the intestines of the treated mice, while a few worms were still present in the intestines of the control, non-treated group.

It was clear that adding MM to the Ag-NPs improved the efficacy of the tested nanoproduct. Supplementing the material proved to have synergistic action against the parasite. However, this was accepted according to Hasan et al.^23^, who mentioned that MM can diminish all stress parameters, mainly oxidative stress. In addition, Namazi et al.^56^ noted that an antioxidant administration combined with Ag-NPs is needed to alleviate its toxicity and allow the body to benefit from its high efficacy. However, the previously described effect of MM can improve mice's general health and immunological response against these parasites.

The absence of adult worms in intestines after 33 dpi was previously mentioned by Khalifa et al.^28^, as this can be explained as a result of the effect of the drugs in addition to the normal life span of the adult worms in the intestinal tissues even in the non-treated mice.

The tested forms of Ag-NPs and albendazole killed the newborn larvae, and early encysted ones were produced during the first 3–8 dpi, significantly decreasing the isolated larvae from the sacrificed mice. However, the obtained larvae failed to infect new mice. However, this agrees with Yadav and Temjenmongla^32^, who stated that the larvae received after 10 days cannot induce infection as *T. spiralis* completes its migration and full development of cysts in 12–14 days. The cysts become infective on the 16th day post-infection.

Testing the product's efficacy against *T. spiralis* encapsulated larvae showed a marked superiority for the effect of Ag-NPs + MM compared to the other tested compounds. Early treatment (3–8 dpi) revealed a significant (*P*-value < 0.001) decrease in the intensity of the cysts in muscles per mouse, with high efficacy (89.7%) in the group treated by Ag-NPs + MM. The mean number of cysts collected from mice treated at 8th dpi was higher than that recorded in those exposed to treatment at 3rd dpi; however, this is due to the ability of developed worms to lay larvae for a little longer (8 days) than the early treated ones. It was important to mention that microscopic examination of larvae extracted from groups treated during the early 3–8 dpi were weak or nonmotile and failed to infect new mice, while that obtained from untreated control groups were live and motile and able to induce infection in newly inoculated mice.

Testing the efficacy of nanoproduct in both forms against the encapsulated larvae by inoculating the drugs at 33rd–35th dpi revealed a significant reduction in the mean number of the encapsulated larvae from 576.4 ± 42.16 in the untreated groups to 255.2 ± 28.33, 243.6 ± 16.52, and 268.8 ± 30.87 in mice treated with 21.5 mg/kg Ag-NPs, 21.5 mg/kg Ag-NPs + MM and 50 mg/kg albendazole, respectively. It was essential to determine that the extracted larvae from the muscles of the groups treated by both forms of Ag-NPs could not infect newly inoculated mice. However, this agrees with García et al.^57^, who demonstrated that encapsulated larvae are more affected than adult worms. At the same time, the larvae extracted from the groups treated with albendazole and those from control untreated mice induced infection in newly inoculated mice. Regarding the incapacity of albendazole and the majority of other trichinocidal drugs to eliminate encapsulated *T. spiralis* larvae, this aligns with the findings reported by El-Wakil et al.^58^ and Abuelenain et al.^59^. These studies noted that adult worms within the intestine exhibit greater susceptibility to drugs than larval cysts in muscles. Concerning the stages of development of the capsule or cyst wall around the migrating larvae in this study, the wall of this cyst appeared thin without complete development to the structure of the nurse cell, and during this, the larvae additionally seemed to be large. During this post-migration period of larvae, as determined by Yadav and Temjenmongla^32^, about 10 days post-larvae migration, most antinematodal drugs can kill these larvae. However, this leads to a reduction in the number of cysts produced. However, after this period, the cyst formation and nurse cell development processes are completed at this stage (encapsulate larvae), and the antinematodal drugs fail to penetrate to kill the larvae. However, this is the new substantial effect obtained by Ag-NPs in the present study, as it was proved to puncture the cyst and kill the late-capsulated larvae. It has not degenerated in the present study, as it was inspected in the treated mice after 3 dpi, but it was present inside its capsule and unable to infect new mice. Collectively, it was demonstrated that Ag-NPs revealed slightly higher (non-significant) efficacy than albendazole at 50 mg/kg (drug control). They showed efficacy against both adult worms and early-migrating muscle larvae. Furthermore, it is crucial to consider the differences in parasite strains in different regions that could affect the acquired results from other studies owing to the parasite genotype effect^60–62^. Other aspects could affect the results in different regions, including the formulation of tested material, doses, and the animal model^63,64^.

Inspecting the alteration of two redox parameters (MDA and TAC) in the muscle and intestine homogenates of *T. spiralis* infected, treated, and control mice revealed that infection by *T. spiralis* significantly disturbed (*P*-value ≤ 0.001) these parameters in comparison with the original level of them in the control non-infected mice. However, this agrees with Othman et al.^36^, who mentioned that infection by *T. spiralis* led to the production of oxidative stress in the hosts. This stress was in the form of excessive production of ROS and other radicals as a defense reaction between the parasite and the host immune mechanism. Concerning MDA and TAC parameters, the alteration level was high in mice exposed to both infection and Ag-NP inoculation. The adverse effects of Ag-NPs on the animal body were previously described by Repetto et al.^22^, who stated that Ag-NPs enhance lipid peroxidation in the liver, making the liver vulnerable to oxidative damage. Furthermore, Moradi-Sardareh et al.^65^ revealed that the Ag-NPs increased lipid peroxidation with a marked increase in serum and tissue MDA levels. However, this is contrary to Ibrahim et al.^66^, who stated that Ag-NPs (175 and 350 ppm orally) reduced the level of MDA, alleviated the oxidative stress and lipid peroxidation with antioxidant properties, and showed gastro-protective effects against ethanol-induced gastric damage in rats.

## Conclusion

Ag-NPs at 21.5 mg/kg B.W. proved to be the only trichinocidal drug that could penetrate and kill the encapsulated larvae and the adult intestinal stage during a very short administration time. Combining Ag-NPs with multivitamins overcame the known drawbacks of silver material and ameliorated its adverse effects on the estimated redox parameters and liver and kidney biomarkers, denoting its ability to alleviate Ag-NPs toxicity. The ability of the product as a trichinocidal drug using a very low dose and a short inoculation period introduced these Ag-NPs as an alternative to anti-helminthic other medicines.

## Data Availability

All data analyzed during this study are included in this published article. The datasets generated during the study are available in the GENE BANK repository, accession numbers: [OR271983].
